# Vasospastic angina in a young female-to-male transsexual

**DOI:** 10.1093/ehjcr/ytaf026

**Published:** 2025-01-23

**Authors:** Miu Eguchi, Yosuke Watanabe, Manabu Uemastu, Akira Sato

**Affiliations:** Department of Cardiology, University of Yamanashi, Shimokato 1110, Chuo, Yamanashi 409-3898, Japan; Department of Cardiology, University of Yamanashi, Shimokato 1110, Chuo, Yamanashi 409-3898, Japan; Department of Cardiology, University of Yamanashi, Shimokato 1110, Chuo, Yamanashi 409-3898, Japan; Department of Cardiology, University of Yamanashi, Shimokato 1110, Chuo, Yamanashi 409-3898, Japan

## Case description

**Figure 1:**
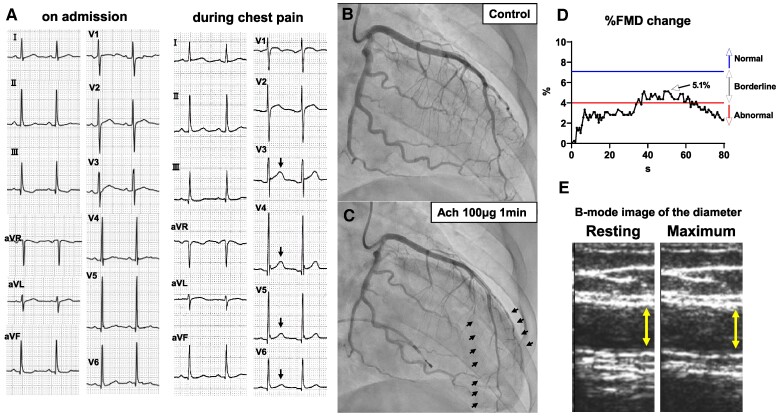
(*A*) Control electrocardiogram on admission (left panel), electrocardiogram during chest pain showed ST elevation in leads V3–6 (arrows) (right panel). (*B*, *C*) Spasm provocation test. Control angiography of the left coronary artery (*B*). Angiography after injection of 100 mg of acetylcholine into the left coronary artery revealed spasm of the distal portion of the left anterior descending artery (arrows) (*C*). (*D*, *E*) The resulting flow-mediated dilation response. Time course of flow-mediated dilation (*D*). Maximum percentage flow-mediated dilation change is 5.1% (arrows). *x*-axis: time (seconds). *y*-axis: percentage flow-mediated dilation change (%). Image of the resting (left panel) and maximum (right panel) flow-mediated dilation test (*E*). FMD, flow-mediated dilation.

A 31-year-old female-to-male transsexual was admitted to our hospital due to several nightly episodes of angina at rest lasting for five minutes. The patient, a current smoker, underwent a bilateral gonadectomy as part of his gender reassignment surgery at age 25 years. The postoperative course was uneventful, and he had received testosterone hormone therapy. His serum testosterone levels (5.95 ng/dL) were higher than that of premenopausal females (normal range, 0.15–0.44 ng/mL), while his estradiol levels (33.1 ng/dL) were comparable with postmenopausal females (normal range, ≦47.0 pg/mL). He experienced chest pain with ST-segment elevation in leads V3–6 on electrocardiogram during the night that he was hospitalized (*Figure 1A*). Coronary angiography revealed no significant arterial stenosis. However, intracoronary administration of 100 μg of acetylcholine during a spasm provocation test induced a spasm in the left anterior descending artery (*Figure 1B* and *C*). Flow-mediated dilation (FMD) of the brachial artery showed decreased endothelial function with impaired FMD (5.1%) (*Figure 1D* and *E*). He was diagnosed with vasospastic angina, and oral administration of 100 mg diltiazem improved his symptoms.

The early-onset vasospastic angina is rare, especially in females. A higher testosterone/estradiol ratio in females was associated with an elevated risk for cardiovascular disease.^1^ This case suggests that bilateral gonadectomy and subsequent testosterone therapy, as part of gender reassignment, may increase the risk of early-onset vasospastic angina and systemic endothelial dysfunction. In postmenopausal women with vasospastic angina, oestrogen replacement therapy may be considered; however, in this case, calcium channel blockers and nitrates are considered the preferred treatment.

Microvascular dysfunction is also associated with declined oestrogen levels^2^ and vasospastic angina.^3^ A history of gender reassignment may therefore predispose individuals not only to vasospastic angina but also to microvascular dysfunction.

To our knowledge, this is the first report of vasospastic angina in a young female-to-male transgender individual.

**Consent:** Written informed consent was obtained from the patient for the publication of this case report and associated images.

**Funding:** None declared.

## Data Availability

The data underlying this article are available in the article.
